# EpCAM^low^ Circulating Tumor Cells: Gold in the Waste

**DOI:** 10.1155/2019/1718920

**Published:** 2019-09-17

**Authors:** Chiara Nicolazzo, Angela Gradilone, Flavia Loreni, Cristina Raimondi, Paola Gazzaniga

**Affiliations:** ^1^Department of Molecular Medicine, Sapienza University of Rome, 00161 Rome, Italy; ^2^Department of Radiological, Oncological and Anatomopathological Sciences, Sapienza University of Rome, 00161 Rome, Italy

## Abstract

The CellSearch® system which is still considered the gold standard for the enumeration of circulating tumor cells (CTC) utilizes antibodies against the epithelial cell adhesion molecule (EpCAM) for CTC enrichment. Recently, CTC discarded by the CellSearch® system due to their low EpCAM expression have been isolated and analyzed. We here sought to discuss technical and biological issues concerning the isolation and characterization of EpCAM^low^ CTC, highlighting the enormous potential of this subpopulation discarded by CellSearch®, which might instead reveal an unexpected clinical significance in tumor types where CTC enumeration has never been validated for prognostic and predictive purpose.

## 1. Introduction

The CellSearch® system (Menarini Silicon Biosystems, Castel Maggiore, BO, Italy) was placed on the market by Veridex Corporation (Warren, NJ) in 2004, and despite 15 years having passed, it still considered the gold standard for the enumeration of circulating tumor cells (CTC), the first and the only one cleared by the US FDA for monitoring of metastatic breast, colorectal, and prostate cancers [1].

The CellSearch® system detects CTC from the whole blood of cancer patients through an immunomagnetic selection using a ferrofluid capture reagent. This is a suspension of magnetic nanoparticles conjugated to a mouse monoclonal antibody recognizing the epithelial cell adhesion molecule (EpCAM) present on the surface of epithelial origin cells. The enriched cells are then labelled with fluorescent dyes for the detection of nucleus; cytokeratins (CK) 8, 18, and 19 (as markers of epithelial origin); and CD45 (expressed on leukocytes), to discriminate the cells of epithelial origin from unwanted blood ones [2]. Therefore, an object is defined as a CTC when having round to oval morphology, a visible nucleus, positive staining for CK, and negative staining for CD45, according to the manufacturer's definition [3].

The decision to target an epithelial cell antigen for immunomagnetic enrichment of CTC relies on the premise that epithelial cells are absent into bloodstream under physiological conditions [4]. Based on the evidence that monoclonal antibodies directed against EpCAM are broadly reactive with the tissue of epithelial-derived cancers [5], a series of preliminary studies was performed using flow cytometry assay therefore resulting in the choice of EpCAM as the preferential target for CTC immunomagnetic detection [1]. Nevertheless, in the following years, it became clear that higher numbers of CTC can be detected using alternative, EpCAM-independent methods, suggesting that a mixture of EpCAM-positive and EpCAM-negative tumor cells circulates in the blood [6].

In this review, we will argue the unresolved issue of CTC undetected by CellSearch®, with a particular focus on the latest developments reported by the group of Terstappen. In particular, we will discuss technical and biological issues concerning the isolation and characterization of CTC expressing no or low EpCAM, highlighting the enormous potential of this subpopulation discarded by the system, which might instead reveal an unexpected clinical significance in tumor types where CTC enumeration has never been validated for prognostic and predictive purpose.

## 2. EpCAM^high^ and EpCAM^low^ Circulating Tumor Cells

The presence of CTC exhibiting different phenotypes in the same patient due to tumor heterogeneity induced Terstappen and Co. to conduct in-depth studies on CTC detection through the CellSearch® system, with a focus on discarded ones expressing no or low EpCAM [7–9]. In 2015, the authors described a method to investigate the presence of two subpopulations of CTC: EpCAM^high^ and EpCAM^low^ CTC. After immunomagnetic depletion of EpCAM^high^ cells, the blood sample discarded by CellSearch® was collected through the Automatic Sample Collection Device (ASCS), inserted between the waste tube from CellTracks Autoprep system and the waste container [7]. The discarded blood coming out of the Autoprep was alternatively collected manually by placing a 50 mL conical tube under the outlet [8]. Both ways, the blood sample waste was then passed through the filtration device and the EpCAM^low^ CTC collected on the microsieve were analyzed by immunofluorescence staining [7–9]. A cocktail of fluorescently labeled antibodies (pan-CK and CD45) was used to stain cells and to correctly classify them as CTC. The EpCAM^low^ cells had a nucleus identified by DAPI, expressing CK, but not CD45.

Using such proven and relevant testing protocols and tools, three studies were carried out to address how many CTC showing no or low EpCAM expression were discarded during immunomagnetic isolation by CellSearch® and whether their presence was associated with clinical outcome. Results from a pilot study in patients with metastatic lung cancer did not show any significant correlation between the presence of EpCAM^low^ CTC and overall survival (OS), although the percentage of CTC patients increased when adding the number of CTC found in the blood waste [7]. Similar observations were made in a study involving 97 patients with advanced non-small-cell lung cancer [8]. In 2018, the presence of EpCAM^high^ and EpCAM^low^ CTC was determined in castration-resistant prostate cancer and metastatic breast cancer patients in a multicenter study. Here, again, it was showed that the presence of EpCAM^low^ CTC was not correlated with poor OS, even though the number of CTC increased when considering both CTC subpopulations [9]. In all these studies, a significant difference was observed for the presence of ≥5 EpCAM^high^ CTC in relation to OS whereas no significant difference was observed for ≥5 EpCAM^low^ CTC, demonstrating that the strong correlation with survival can be solely contributed to EpCAM^high^ CTC.

## 3. EpCAM^low^ CTC Isolation. Technical and Biological Issues

Since CellSearch® was designed for the immunomagnetic enrichment and fluorescent labeling in order to detect circulating epithelial-derived tumor cells [10], it seems obvious that any variation in epithelial antigen expression compromises the ability of the system to isolate and to identify CTC. Since a downregulation of CK8/18/19 might lead to a reduced ability of CellSearch® to detect CTC, the addition of antibodies covering a broad spectrum of CK into user-defined marker channel has been demonstrated to be a useful tool to improve CTC detection [7, 8]. Therefore, the main technical issue in CTC count by the CellSearch® system is linked to the dynamic expression of EpCAM, resulting, of course, in a significant reduction in CTC yield [11, 12].

The inability of CellSearch® to isolate EpCAM-negative cells led to a number of studies aimed at comparing the performance of CellSearch® with that of other EpCAM-dependent and EpCAM-independent technologies [7, 10], until Terstappen and Co. for the first time managed CellSearch® instrument in order to recover, isolate, and characterize the cells discarded by the system through the abovementioned approach [7–9].

Before proceeding with patient sample analysis, a series of spiking experiments using cells from tumor cell lines with different EpCAM densities was performed in order to validate the test [7, 8]. The obtained results showed that the cell recovery with CellSearch® was directly related to EpCAM antigen density [7]. Therefore, the system was able to detect the cells expressing relatively high EpCAM, while the EpCAM^low^ cells were mostly recovered in the discarded blood samples. The test was then performed on patient specimens, and there again, cells were found in the blood discarded by the CellSearch® [7–9]. The authors assumed these cells as having no or low EpCAM expression based on data obtained from spiking experiments [7]. Given that the secondary cell lines derive from an already established primary culture and grow in an artificially controlled environment, they are not representative of the high degree of heterogeneity of CTC. In this context and to demonstrate that CTC discarded by CellSearch® really express no or low EpCAM, a simple immunofluorescence staining for EpCAM performed on CTC isolated from blood waste, employing the same clone (VU1D9) used as the system capture reagent, appears to be a necessary step. In our previous study, we demonstrated that 89% of CTC isolated from colorectal cancer patients expressed EpCAM, despite CellSearch® being able to detect only a very small fraction [13]. Furthermore, to corroborate that CellSearch® detects EpCAM^high^ cells rather than EpCAM^low^, it would be interesting to determine the intensity value of EpCAM staining as it has been done for cytokeratins [8]. According to the steric hindrance of CellSearch® capture reagent, we suggest that staining EpCAM using an antibody recognizing an epitope closer to the plasma membrane could allow to evaluate the high and low expression levels of EpCAM in both enriched and discarded CellSearch® cells. It is clear that spiking experiments are necessary in order to verify the feasibility of such approach, since it could not be easy to be performed using CellSearch®.

The basic concern regarding the CellSearch® is represented by its heavy reliance on cell surface expression of EpCAM and, therefore, by the loss of a significant proportion of CTC not having sufficient epithelial cell surface and being thus excluded from the analysis [6]. From a biological point of view, the system inefficiency is generally attributed to epithelial mesenchymal transition (EMT), a precise process which leads a polarized epithelial cell to undergo multiple biochemical changes allowing it to assume a mesenchymal cell phenotype, with distinct and specific features [14]. EMT confers on tumor cell migratory and invasive properties required for the metastatic process [15], thereby downregulating EpCAM expression. However, it has been demonstrated that EMT is not always required for metastasis [16, 17] and evidence of hybrid CTC coexpressing both epithelial and mesenchymal markers which are attributed to them enhanced metastatic potential has been also provided [18]. Moreover, it is conceivable that the presence of EpCAM^high^ CTC is due to highly proliferative epithelial cells which prevail over mesenchymal ones in generating macrometastasis [19]. On the other hand, mechanisms, other than EMT, might be involved in spreading of EpCAM^low^ CTC [17]. Conditions of profound stress, such as treatment with antitumor agents and hypoxia, promote cellular plasticity without necessarily involving the loss of EpCAM. For instance, in an *in vitro* study, the chronic exposure to bevacizumab induced underestimation of CTC through CellSearch® due to altered EpCAM isoform expression [20]. Different conformational states of the antigen might mask the antibody binding site or even decrease its binding affinity, especially when EpCAM is immobilized on magnetic core nanoparticles and thus its orientation is limited by steric hindrance. Furthermore, the proteolytic cleavage containing the epitope recognized by EpCAM clone might negatively affect CTC detection [13].

## 4. EpCAM^low^ CTC from a Clinical Perspective

Overall, numerous studies have provided evidence that CTC expressing no or low EpCAM are frequently detected in specific tumor types [21–25] and an increasing number of studies have demonstrated that EpCAM-negative CTC are associated to worse prognosis. EpCAM-negative CTC, thus undetected through CellSearch®, have been correlated with poor prognosis in colorectal cancer patients in the course of antiangiogenic treatment [26] as well as with the emergence of brain metastasis in triple-negative breast cancers [27]. Further studies using multiparametric flow cytometry have associated EpCAM-negative CTC with a significantly decreased OS in breast cancer patients [28]. CTC negative for EpCAM, but expressing different stem-like markers, were found directly involved in increased brain metastatic properties using breast cancer and lung cancer *PDX* models [29]. EpCAM^low^ CTC have been found significantly associated with tumor depth, lymph node metastasis, and increased malignancy in gastric and endometrial cancers after staining by anti-CD45, anti-EpCAM, anti-CK, and anti-CEA (CD66e) antibodies [30, 31]. All these studies suggest that EpCAM^low^ cells have prognostic significance independent of the isolation method used. Furthermore, evidence has been provided that EpCAM-negative CTC are preferentially associated to brain metastases [32], while EpCAM-expressing CTC are more prognostic in tumors with propensity to bone metastases [33], thus suggesting that EpCAM-positive and EpCAM-negative subpopulations of CTC are able to differently determine organ tropism [34]. In this context, the results obtained by de Wit et al., who investigated the prognostic role of EpCAM-depleted blood fractions in prostate and breast cancers, could be better explained [8]. Authors failed to demonstrate any prognostic value of EpCAM^low^ CTC for OS in castration-resistant prostate and breast cancer patients, without taking into consideration that they analyzed only patients with tumors characterized by propensity to bone metastases. Alternatively, the choice to detect EpCAM^low^ CTC using pan-CK antibodies, thus missing pure mesenchymal CTC in the EpCAM^low^ cell fraction, might be a further explanation of their results. Indeed, several studies have demonstrated that pure mesenchymal CTC phenotypes are associated with inferior prognosis regardless of EpCAM expression [35–37]. Thus, the prognostic and predictive significance of EpCAM^low^ CTC still leaves many open questions and the reasons for contradictory results between studies should be urgently addressed. The widely accepted concept of intratumoral heterogeneity, which represents the lifeblood of cancer, implies that this heterogeneity must be necessarily maintained at the CTC level. From this point of view, the presence of distinct CTC pools characterized by different EpCAM expressions is not surprising. Although the prognostic and predictive value of EpCAM^high^ CTC has been widely demonstrated at least in some tumor types, no studies comparing the EpCAM^high^ and EpCAM^low^ subpopulations isolated from the same patient have been provided to date [38]. At this regard, the study by de Wit et al. sounds pioneering in that it would allow to evaluate the prognostic and predictive potential of enriched CTC as well as those discarded with the same method, in the same patient, in the same blood sample, and in a single workflow. It is conceivable that the isolation and characterization of CTC discarded by CellSearch®, due to the low expression of EpCAM, might reveal an unexpected clinical significance especially in those tumor types where CTC enumeration has never been validated for prognostic and predictive purpose.

## References

[1] Swennenhuis J. F., van Dalum G., Zeune L. L., Terstappen L. W. M. M. (2016). Improving the CellSearch® system. *Expert Review of Molecular Diagnostics*.

[2] Coumans F., Terstappen L. (2015). Detection and characterization of circulating tumor cells by the CellSearch® approach. *Methods in Molecular Biology*.

[3] Allard W. J., Matera J., Miller M. C. (2004). Tumor cells circulate in the peripheral blood of all major carcinomas but not in healthy subjects or patients with nonmalignant diseases. *Clinical Cancer Research*.

[4] Riethdorf S., O'Flaherty L., Hille C., Pantel K. (2018). Clinical applications of the CellSearch® platform in cancer patients. *Advanced Drug Delivery Reviews*.

[5] Moreno J. G., O’Hara S. M., Gross S. (2001). Changes in circulating carcinoma cells in patients with metastatic prostate cancer correlate with disease status. *Urology*.

[6] Agnoletto C., Minotti L., Brulle-Soumare L. (2018). Heterogeneous expression of EPCAM in human circulating tumour cells from patient-derived xenografts. *Biomarker Research*.

[7] de Wit S., van Dalum G., Lenferink A. T. M. (2015). The detection of EpCAM^+^ and EpCAM^−^ circulating tumor cells. *Scientific Reports*.

[8] de Wit S., Manicone M., Rossi E. (2018). EpCAM^high^ and EpCAM^low^ circulating tumor cells in metastatic prostate and breast cancer patients. *Oncotarget*.

[9] Wit S., Rossi E., Weber S. (2019). Single tube liquid biopsy for advanced non‐small cell lung cancer. *International Journal of Cancer*.

[10] Andree K. C., van Dalum G., Terstappen L. W. M. M. (2016). Challenges in circulating tumor cell detection by the CellSearch® system. *Molecular Oncology*.

[11] Gires O., Stoecklein N. H. (2014). Dynamic EpCAM expression on circulating and disseminating tumor cells: causes and consequences. *Cellular and Molecular Life Sciences*.

[12] Raimondi C., Nicolazzo C., Gradilone A. (2014). Circulating tumor cells: exploring intratumor heterogeneity of colorectal cancer. *Cancer Biology & Therapy*.

[13] Nicolazzo C., Raimondi C., Francescangeli F. (2017). EpCAM-expressing circulating tumor cells in colorectal cancer. *The International Journal of Biological Markers*.

[14] Kalluri R., Weinberg R. A. (2009). The basics of epithelial-mesenchymal transition. *The Journal of Clinical Investigation*.

[15] Gloushankova N. A., Zhitnyak I. Y., Rubtsova S. N. (2018). Role of epithelial-mesenchymal transition in tumor progression. *Biochemistry (Mosc)*.

[16] Diepenbruck M., Christofori G. (2016). Epithelial-mesenchymal transition (EMT) and metastasis: yes, no, maybe?. *Current Opinion in Cell Biology*.

[17] Le Magnen C., Shen M. M., Abate-Shen C. (2018). Lineage plasticity in cancer progression and treatment. *Annual Review of Cancer Biology*.

[18] Jolly M. K., Mani S. A., Levine H. (2018). Hybrid epithelial/mesenchymal phenotype(s): the ‘fittest’ for metastasis?. *Biochimica et Biophysica Acta (BBA) - Reviews on Cancer*.

[19] Fischer K. R., Durrans A., Lee S. (2015). Epithelial-to-mesenchymal transition is not required for lung metastasis but contributes to chemoresistance. *Nature*.

[20] Nicolazzo C., Massimi I., Lotti L. V. (2015). Impact of chronic exposure to bevacizumab on EpCAM-based detection of circulating tumor cells. *Chinese Journal of Cancer Research*.

[21] Bulfoni M., Turetta M., del Ben F., di Loreto C., Beltrami A., Cesselli D. (2016). Dissecting the heterogeneity of circulating tumor cells in metastatic breast cancer: going far beyond the needle in the haystack. *International Journal of Molecular Sciences*.

[22] Kelley R. K., Magbanua M. J. M., Butler T. M. (2015). Circulating tumor cells in hepatocellular carcinoma: a pilot study of detection, enumeration, and next-generation sequencing in cases and controls. *BMC Cancer*.

[23] Gorin M. A., Verdone J. E., van der Toom E., Bivalacqua T. J., Allaf M. E., Pienta K. J. (2017). Circulating tumour cells as biomarkers of prostate, bladder, and kidney cancer. *Nature Reviews Urology*.

[24] Chalfin H. J., Kates M., van der Toom E. E. (2018). Characterization of urothelial cancer circulating tumor cells with a novel selection-free method. *Urology*.

[25] Lindgren G., Wennerberg J., Ekblad L. (2017). Cell line dependent expression of EpCAM influences the detection of circulating tumor cells with CellSearch. *Laryngoscope Investigative Otolaryngology*.

[26] Gazzaniga P., Raimondi C., Gradilone A. (2011). Circulating tumor cells, colon cancer and bevacizumab: the meaning of zero. *Annals of Oncology*.

[27] Mego M., De Giorgi U., Dawood S. (2011). Characterization of metastatic breast cancer patients with non-detectable circulating tumor cells. *International Journal of Cancer*.

[28] Lustberg M. B., Balasubramanian P., Miller B. (2014). Heterogeneous atypical cell populations are present in blood of metastatic breast cancer patients. *Breast Cancer Research*.

[29] Bertolini G., D'Amico L., Moro M. (2015). Microenvironment-modulated metastatic CD133+/CXCR4+/EpCAM- lung cancer-initiating cells sustain tumor dissemination and correlate with poor prognosis. *Cancer Research*.

[30] Miki Y., Yashiro M., Okuno T. (2017). Clinical significance of EpCAM-negative and CEA-positive circulating tumor cells in gastric carcinoma. *Cancer Research*.

[31] Wen K. C., Sung P. L., Chou Y. T. (2018). The role of EpCAM in tumor progression and the clinical prognosis of endometrial carcinoma. *Gynecologic Oncology*.

[32] Hanssen A., Riebensahm C., Mohme M. (2018). Frequency of circulating tumor cells (CTC) in patients with brain metastases: implications as a risk assessment marker in oligo-metastatic disease. *Cancers (Basel)*.

[33] Fu L., Zhu Y., Jing W. (2018). Incorporation of circulating tumor cells and whole-body metabolic tumor volume of 18F-FDG PET/CT improves prediction of outcome in IIIB stage small-cell lung cancer. *Chinese Journal of Cancer Research*.

[34] Micalizzi D. S., Maheswaran S., Haber D. A. (2017). A conduit to metastasis: circulating tumor cell biology. *Genes & Development*.

[35] Aktas B., Tewes M., Fehm T., Hauch S., Kimmig R., Kasimir-Bauer S. (2009). Stem cell and epithelial-mesenchymal transition markers are frequently overexpressed in circulating tumor cells of metastatic breast cancer patients. *Breast Cancer Research*.

[36] Mego M., Mani S. A., Lee B. N. (2012). Expression of epithelial-mesenchymal transition-inducing transcription factors in primary breast cancer: the effect of neoadjuvant therapy. *International Journal of Cancer*.

[37] Yokobori T., Iinuma H., Shimamura T. (2013). Plastin3 is a novel marker for circulating tumor cells undergoing the epithelial-mesenchymal transition and is associated with colorectal cancer prognosis. *Cancer Research*.

[38] Krebs M. G., Hou J. M., Ward T. H., Blackhall F. H., Dive C. (2010). Circulating tumour cells: their utility in cancer management and predicting outcomes. *Therapeutic Advances in Medical Oncology*.

